# A Preliminary Evaluation of Sex and Dietary Field Pea Effects on Sensory Characteristics of Dry-Cured Loins

**DOI:** 10.3390/ani14050739

**Published:** 2024-02-27

**Authors:** Immaculada Argemí-Armengol, Javier Álvarez-Rodríguez, Marc Tor, Laura Salada, Ana Leite, Lia Vasconcelos, Alfredo Teixeira, Sandra Sofia Quinteiro Rodrigues

**Affiliations:** 1 Department of Animal Science, University of Lleida, 25198 Lleida, Spain; javier.alvarez@udl.cat (J.Á.-R.); marc.tor@udl.cat (M.T.); laura.saladaherms1@gmail.com (L.S.); 2 Centro de Investigação de Montanha (CIMO), Instituto Politécnico de Bragança, Campus de Santa Apolónia, 5300-253 Bragança, Portugal; anaisabel.leite@ipb.pt (A.L.); lia.vasconcelos@ipb.pt (L.V.); teixeira@ipb.pt (A.T.); srodrigues@ipb.pt (S.S.Q.R.); 3 Laboratório para a Sustentabilidade e Tecnologia em Regiões de Montanha, Instituto Politécnico de Bragança, Campus de Santa Apolónia, 5300-253 Bragança, Portugal

**Keywords:** castration, immunocastration, diet, skatole, pork dry-cured loins, sensory evaluation, taste panel, consumers

## Abstract

**Simple Summary:**

This preliminary study investigated the sensory quality perceived by consumers (trained panellists or untrained consumers) of pork loins deriving from animals subjected to different castration methods (surgical vs. immunocastration) and fed different protein sources (soybean vs. pea). The findings revealed that the trained panel could discriminate clearly between dry-cured loin from male pigs surgically castrated and fed a soya diet compared with those fed a pea diet, and the vaccination almost completely reduced most of the sensory traits in both sexes. Additionally, the study found that immunocastration in male pigs did not negatively impact the skatole detection scoring. Moreover, the untrained consumer panel preferred the dry-cured loin from surgically castrated male pigs fed a pea-based diet. To sum up, it is possible to completely replace soybean meal with field peas when feeding heavy immunocastrated male pigs for dry-cured loin production.

**Abstract:**

Two of the main issues related to cured meat products are castration to avoid boar taint and the hefty reliance on soybean meal to feed pigs. However, data on the effects of immunocastration in pigs and alternative crop protein feeds on the sensory traits and consumers’ acceptance of dry-cured loin are still limited. A preliminary study was conducted on the effect of sex type (surgical castrated male pigs and immunocastrated male and female pigs, at approximately 140 kg in weight and 7 months of age) and animal diet (pea vs. soya) on dry-cured loins. The study involved a sensory evaluation of six treatments, with a 3 × 2 factorial design, conducted by trained panellists and untrained consumers in Spain (*n =* 126) and Portugal (*n =* 80). The consumers were also checked for their skatole sensitivity using a pure substance. The results showed that the pea-based diet was significantly different (*p* < 0.001) from the soy-based diet, as determined by a trained panel of surgically castrated male pigs, although the immunocastration treatments were not split. Dry-cured loin from immunocastrated male pigs fed with peas was considered tenderer and juicier (*p* < 0.05) than those fed soya-based diets. The untrained consumer panel scored higher on the sensory traits (flavour, juiciness, and overall liking) from the loin samples of surgically castrated male pigs fed a pea diet. All consumers scored similarly in boar taint detection. This study demonstrates the applicability of the pea-based diet for the feeding of pigs destined for cured meat and highlights immunocastration as a tool that does not compromise the skatole detection score.

## 1. Introduction

The quality of dry-cured pork loins can be influenced by various factors such as genetics, feed, and rearing conditions. These factors play a crucial role in determining consumer preferences and satisfaction as they effect the sensory characteristics of the food. The dry-cured loin is a popular product consumed worldwide and is made from one of the most valuable muscle cuts of the pork carcass (*Longissimus thoracis et lumborum* (LTL) muscle) [1]. Food traditions and cultural practices have an impact on customer eating behaviours and preferences [2]. Sensory analysis is a method used to evaluate food properties through the senses. The methodology used to construct sensory profiles by trained and untrained panellists differs between them, either scoring of attributes or preference for meat [3].

Soybean meal is the main protein source used in animal feed [4]. The imbalance between demand and supply increases the price of soy and its by-products. The main producers of soybean 2022/2023 were Brazil, Argentina, and the United States [5]. However, its cultivation leads to deforestation [6]. This has resulted in great pressure to develop governance initiatives to increase the EU production of plant-based proteins as a means to reduce its protein import dependency and reduce food prices [7].

The Berkshire breed has excellent meat quality characteristics, such as thin muscle fibre and an excellent water-holding capacity, whereas the Duroc breed is used as a terminal sire when fattening pigs are produced, due to its excellent growth rate and much higher amount of intramuscular fat [8]. Crossbreeding is aimed to increase overall efficiency. Producing heavy pigs, greater than 130 kg of weight, aims to provide high conformed cuts and fat accretion, to produce high-quality processed products [9].

A widespread ancient practice is to castrate male piglets to prevent boar taint. Boar taint is an offensive odour and taste that can be evident during cooking or eating pork, primarily caused by skatole and androstenone. Also, castration is used to increase carcass fat content. However, the European pig industry sector has considered abandoning this practice for animal welfare issues [10]. Skatole (3-methylindole) is produced by both male and female pigs from the microbiological degradation of dietary L-tryptophan in the hindgut. Many studies have shown negative impacts on meat quality and consumer reactions to skatole and boar taint in meat [11]. An alternative to surgical castration is immunization against GnRH (vaccination) in both male and female pigs. This alternative can influence the hormonal turnover and the animals’ meat quality [12].

The main objective of this study was to evaluate the sensory perception of both trained panellists and consumers, in order to identify any potential differences in dry-cured loins. Specifically, the study aimed to determine if there were any differences based on of the sex type and castration technique (castrated male pigs, immunocastrated male and female pigs), as well as legume diet (soya and pea). The study used a 3 × 2 factorial experiment. Additionally, the study sought to determine the skatole sensitivity of untrained consumers across various regions with different consumption patterns of pork, through an olfactory test.

## 2. Materials and Methods

As a preliminary study, six different treatments and two tests (trained panellists and a consumer panel) were conducted to investigate the impact of the castration type and pig sex, as well as the possibility of substituting whole soya with field peas in late fattening pig diets (for pigs weighing 110–140 kg) on the quality of dry-cured loins. The first test involved a trained taste panel, which examined different sensory descriptors in dry-cured loin treatments and evaluated their sensitivity to boar taint, including odour. The second test involved an untrained consumer panel, conducted in two countries: Spain (with high pork consumption) and Portugal (with slightly lower consumption). In this test, a sensory analysis was carried out to evaluate the preference of consumers for different treatments. The consumers panel also studied the sensitivity to skatole at different concentrations.

### 2.1. Experimental Design

The material used in this trial was obtained from the same pigs as those used in a previous experiment detailing the technological quality and fatty acid profile in meat [13]. The experimental design consisted of a factorial design with three sex categories, i.e., immunocastrated males (IM), surgical castrated male pigs (CM), and immunocastrated female pigs (F), and two animal diets during the entire fattening period (from 40 kg to 140 kg of body weight), involving the control (business-as-usual outsourced soybean meal as the main amino acid source, S) and experimental diet (field pea seeds, *Pisum sativum*-locally grown, P). Pea was gradually included at 25% (40–80 kg), 30% (80–110 kg), and finally 40% (110–140 kg), with partial to entire replacement of soybean meal in feed. This was performed gradually to allow adaptation to the increased digestive tract capacity of the pigs. The experimental design was completely randomized. Loins came from one hundred and twenty pigs (crossbreds of Duroc sows with Berkshire boars) raised on the same farm (Artés, Barcelona, Spain), using twenty loins from pigs for each treatment randomly. Pigs were slaughtered in a single batch and were of similar age (~218 days) and weight (~143 kg) at slaughter. Immunocastrated male and female pigs received two applications of the vaccines Improvac^®^ and Vacsincel^®^ (Zoetis, Zaventem, Belgium), respectively, according to the manufacturer’s recommendations. Fresh pork loins were collected 2 h postmortem (*n =* 20 per treatment) and refrigerated in a chamber between 2 and 5 °C.

### 2.2. Dry-Curing Process and Sampling

The loins (approximately 3 kg weight each) were transported to be processed at the commercial plant Catalana d’Embutidos, S.A. (Balsareny, Barcelona, Spain). The surface fat and connective tissue were removed, and loins were marinated for two weeks in a big box (4 °C and relative humidity 75% to 80%). At this stage, the loins were placed in a rotating drum for salting and seasoning (added mixture before). The marination ingredients were salt, lactose, red pepper, spices, dextrose, and additives: flavour enhancers (E-621), preservatives (E-252, E-250), and antioxidants (E-301). Afterwards, loins were brushed to remove the excess seasoning mixture and stuffed into collagen casings. They were held for approximately 9 weeks at 9 °C and relative humidity of 80% to 85% to be cured. Once dried and cured, the loins were vacuum-packaged and kept at 4 °C. Five loins from each treatment were randomly selected (total number of samples = 30) for sensory analyses of the trained panel and the consumers’ assessment.

### 2.3. Sensory Analysis

Three loins randomly selected out of six treatments (eighteen loins in total) were used by the trained panel and consumer panel in Bragança (Portugal), and two loins at random (twelve loins in total) were assessed by the sensory evaluation of consumer panel in Lleida (Spain). Dry-cured loin samples (approximately 100–120 mm in diameter) were cut (1.5 mm thickness) and sliced with a cut machine in each sensory analysis session.

#### 2.3.1. Trained Panel

A trained panel of eight experts performed a sensory analysis in three sessions on different days, already recruited for other research studies with meat and trained specifically to taste pork meat, according to the Portuguese Standard (NP-ISO-8586-1, 2001) [14]. Training consisted of two phases and two sessions. The first is based on an individual evaluation of dry-cured loin samples from different manufacturers, and the second was open discussion to adapt the panel elements to scales and sensory descriptors. After training the panellists and agreeing on the most suitable features to evaluate dry-cured loin, the visual appearance was assessed based on four parameters (grading scores 1 to 9): muscle colour of lean cured meat (pale rose (on left) to dark brown (on the right)); fat colour (intensity of white (on the left) to yellow colour (on the right) on fat); muscle/fat ratio (fat to muscle dominance, fatness predominates (on the left) to leanness predominates (on the right)); and fat distribution (homogenous (on the left) to localized (on the right)). The texture perceived in the mouth was evaluated against three parameters (grading scores 1 to 9): hardness (perception of firmness during chewing, tenderness (on the left) to toughness (on the right)); chewiness (chewing facility, easy (on the left) to difficult (on the right)); and juiciness (impression of lubricated food during chewing, from dry (on the left) to juicy (on the right)). The intensity of flavours was also evaluated using six parameters, ranging from low (on the left) to high (on the right) (grading scores 1 to 9): saltiness, sweetness, acidity, bitterness, flavour persistence, and flavour intensity (overall assessment). Subsequently, the curing aroma was evaluated through parameter odour intensity ranging from low (on the left) to high (on the right) (grading scores 1 to 9). Finally, during the sensory assessment, the panellists were asked if a strange odour was detected, in addition to scoring the description of boar taint odour by two parameters, ranging from none (on the left) to high (on the right): detection of urine odour (androstenone) and detection of manure/stable odour (skatole).

Afterwards, sensory evaluations were conducted in a specific tasting room of the Polytechnic Institute of Bragança. During evaluations, room temperature and relative humidity were maintained at 20–22 °C and 50–60%. The room light was white, and, in each booth, red light was used to mask the sample colour. Each panellist was provided with mineral water and plain crackers to clear their palate in between samples. Then, samples were cut (1.5 mm thickness) and sliced with a cutting machine (Fac Type F250E, Isola Vicentina, Italy), and they were presented to each sensory analysis panellist in the same conditions, blind, in a random and balanced distribution order, coded with 3-digit numbers. The sensory profile, qualitative and quantitative appearance, odour, texture, and taste attributes were evaluated in the cured loins (16 descriptors). All treatments were evaluated in duplicate in each of the 3 sessions. In each session, panellists evaluated 12 samples (6 samples at 11 am and 6 samples at 4 pm) using a scale with intervals numbered. The methodology used was described previously by Tolentino [15] and by the Portuguese Standards [14]. Panellists were asked to evaluate on the scale corresponding to the intensity of their different feelings for each attribute.

#### 2.3.2. Consumer Panel

Consumers from Lleida in Spain (at the University of Lleida School of Agrifood and Forestry Engineering and Veterinary Medicine, ETSEAFIV) (*n =* 126) and Bragança in Portugal (Agriculture School of Polytechnic Institute of Bragança, IPB-ESA) (*n =* 80) were enrolled to evaluate dry-cured loins. In Lleida and Bragança, the loins were sliced (1.5 mm thickness) by machine cutting (Romagsa, AF 350 FR-INGR, Ripollet, Spain; and Fac, F250E, Isola Vicentina, Italy, respectively). Participants were asked to complete a survey with 4 terms related to the sensory characteristics of the dry-cured loins, selected based on previous works. They were students and workers who previously received an Instagram message and agreed to taste and evaluate dry-cured loin. The consumers’ perception of dry-cured loin was conducted face to face by trained moderators in each country. The protocol was the basis for the taste session and the procedures were made deeper by the exchange of information and necessary clarifications with moderators during the panel. Previously, a short introduction was given to the consumers about the type of product to be tested (dry-cured loin) and the practical procedure for testing. The following information was given when serving samples: “We will now introduce you to different types of dry-cured loins and we want you to evaluate the sensory attributes to describe the loins, regarding appearance, odour, flavour/taste, and texture (mouthfeel)”. The order of tasting was the decision of the consumer. Four attributes were assessed with a 1 to 5 scale (least (on the left) to best (on the right) score; 5-point facial expression hedonic scale): flavour, crumbliness, juiciness, and overall liking. In addition, the consumers were surveyed concerning some sociodemographic (age and sex), eating habits (dry-cured ham intake pattern), and determinant cues when purchasing dry-cured loins (colour and/or marbling).

The loin pork slices were identified with a 3-digit code and were individually placed on plates that were served to consumers. Between two-sample pork slice tasting, the consumers ate toasted bread sticks and drank mineral water. To ensure sensory evaluation of the 6 dry-cured loin treatments, each consumer tasted four dry-cured loin pork slices in two sessions (Spain) or six dry-cured loin pork slices in one only session (Portugal). In Spain, a total of 96 consumers completed the two sensory evaluation sessions, and in Portugal, 80 consumers completed the evaluations. The enrolled consumers were 60.6% women and 39.4% men, and their age range was between 18 and 61 years old. The average consumer age was slightly higher in Spain than in Portugal (26.9 ± 0.60 vs. 23.3 ± 0.43 years old, respectively).

#### 2.3.3. Consumers’ Sensitivity to Skatole

After sensory evaluation of the dry-cured loin pork slices by consumers, a sensitivity test to skatole (3-methyl-indole) was conducted. Each respondent smelt blindly at random 4 filter paper strips that were included with different concentrations of skatole (2 mg L^−1^, 0.5 mg L^−1^, 0.1 mg L^−1^, and 0 mg L^−1^) and ranked them if they perceived an unpleasant odour or not (Yes/No). The filter papers were identified with a 3-digit code. The order of smelling was the decision of the respondent. The reference standard skatole (3-methyVlindole, 99%) was obtained from Thermo Scientific Chemicals (VWR International Eurolab, S.L.U., Llinars del Vallès, Barcelona, Spain). A stock solution was prepared in methanol at a concentration of 2 mg L^−1^. Working solutions were made over a range of 2 mg L^−1^ to 0.1 mg L^−1^ and for a control. The solutions for the two sessions were stored in dark glass bottles to avoid light, at room temperature (18–20 °C).

### 2.4. Statistical Analysis

Generalised Procrustes analysis (GPA), which minimizes the differences between assessors, identifies agreement between them, and summarizes the sets of 3-dimensional data; and the characterisation of the product procedure, to check which characteristics could discriminate between the products more significantly, were performed using the XL-Stat software Addinsoft 2015. The output values obtained from the characterisation of the product procedure of the XLStat software Addinsoft 2015 are the results of the model used, Y = P (product effect) + J (judge effect), based on the work of Husson and Pagès [3].

The sensory variable scores by trained panel were evaluated with a mixed model, including as fixed effects the animal dietary treatment, animal sex, and panellist testing session, as well as their single interactions, while the trained panel was considered as a random effect with the statistical software JMP Pro 16 (SAS Institute, Inc., Cary, NC, USA).

The sensory attribute scores of the consumer panel were evaluated with the same aforementioned software through a standard least squares model, with six treatments (immunocastrated male pigs with a soya diet—IMS, immunocastrated male pigs with a pea diet—IMP, surgical castrated male pigs with a soya diet—CMS, surgical castrated male pigs with a pea diet—CMP, immunocastrated female pigs with a soya diet—FS, and immunocastrated female pigs with a pea diet—FP). Single interactions are not reported in the text as they were non-significant (*p* > 0.05). Additionally, the effects of country location and skatole sensitivity on consumers’ age were evaluated. Values are presented as the least squares means ± standard error of the mean. The level of significance was set at 0.05. Differences between least squares means were assessed with the Tukey test. The associations between skatole sensitivity, sociodemographic variables, and eating patterns were evaluated with contingency tables and the Pearson test. The skatole concentration ranks were grouped into high (2 mg L^−1^), medium (0.1–0.5 mg L^−1^), or undetectable (0 mg L^−1^) for statistical comparisons. The proportion of consumers failing to claim undetectable skatole concentrations was also considered.

## 3. Results and Discussion

It should be noted that this was a preliminary study and the number of replicates per treatment was limited.

### 3.1. Trained Panel

**Figure 1:**
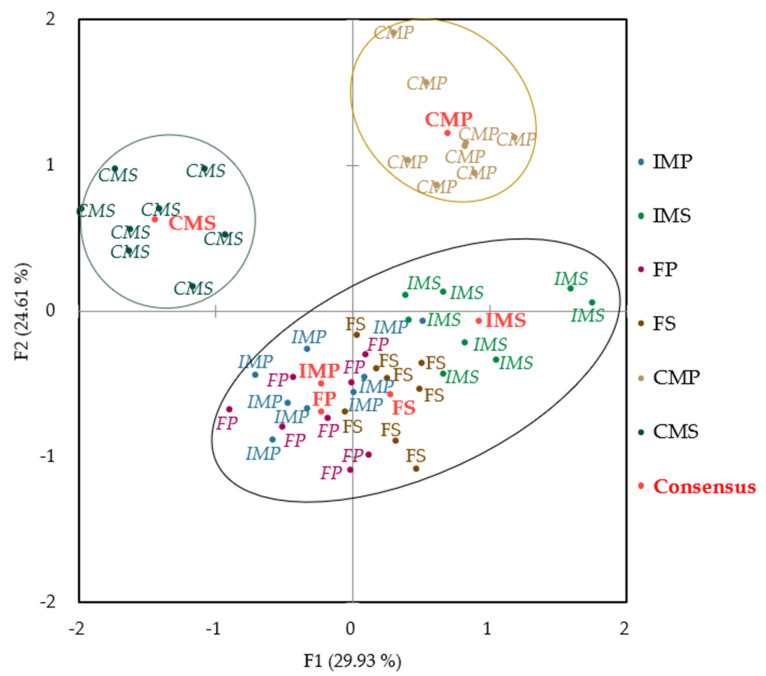
Consensus configuration, by object (different treatments of dry-cured loin). F1 = first principal component of generalized Procrustes analysis (GPA); F2 = second principal component of GPA. IMS = immunocastrated male pigs with a soya diet; IMP = immunocastrated male pigs with a pea diet; CMS = surgical castrated male pigs with a soya diet; CMP = surgical castrated male pigs with a pea diet; FS = immunocastrated female pigs with a soya diet; FP = immunocastrated female pigs with a pea diet.

**Figure 2:**
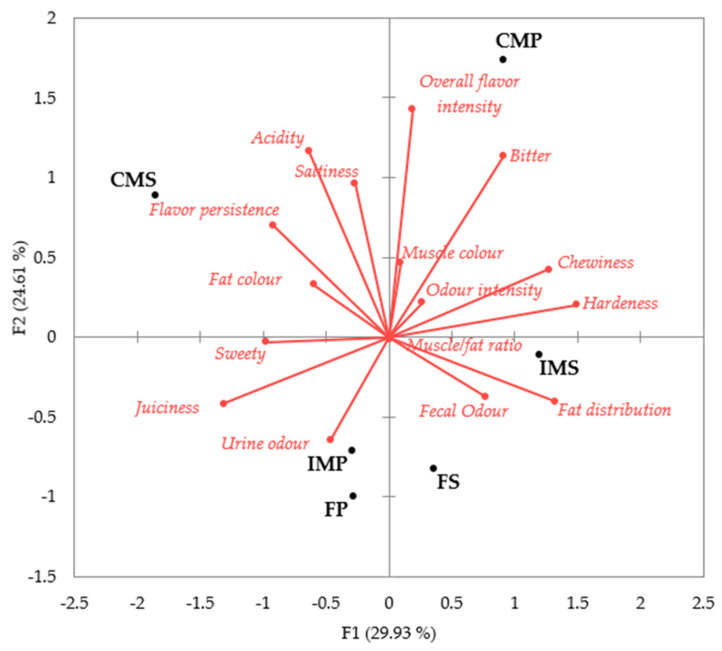
Consensus configuration: joint representation of correlation between sensory attributes (F1 and F2, first and second principal component of generalised procrustes analysis) and the coordinates of the six dry-cured loins treatments analysed: IMS = immunocastrated male pigs with a soya diet; IMP = immunocastrated male pigs with a pea diet; CMS = surgical castrated male pigs with a soya diet, CMP = surgical castrated male pigs with a pea diet; FS = immunocastrated female pigs with a soya diet; FP = immunocastrated female pigs with a pea diet.

The results showed that the panellists P1 and P2 had the highest residual variance, while the panellists P3, P6, P7, and P8 used a wider range of the scale because they had scaling factors higher than 1 (Table 1). The variability between all panellists was explained by the first two main factors (F1 and F2), which accounted for 54.6% of the total variation between samples. Although this value is lower than the 62.5% recorded by Leite et al. [16] in the dry-cured loin of Bisaro pigs, it is still considered a good quality value. In total, five factors were required to explain 100% of the total variability (Table 2). To reduce any differences between the assessors, GPA was used to correct any bias that may have occurred and to reach a consensus (Figure 1 and Figure 2).

A map depicting different treatments of dry-cured loins, categorized by sex types and the animals’ diet, is shown in Figure 1. The majority of the points are situated on the first axis, since around 30% of the variability is concentrated in that area. Additionally, almost all types of dry-cured loin are clearly separated on the map, particularly CMP, CMS, and IMS. While CMS and CMP are separated on the opposite axis of F1 (left and right, respectively), the FP and IMP treatments are located on the left side of F1, whereas the FS and IMS treatments are separated, also on the opposite axis, on the right side of F1. Similarly, surgically castrated pigs are separated on the opposite axis F2 from immunocastrated male and female pigs. This indicates that the panellists could easily differentiate the dry-cured loins from surgically castrated pigs of the two feed types, although they were not able to distinguish as easily between on the other sexes (immunocastrated male and female pigs). This is understandable since immunocastrated males are of intermediate carcass quality compared to surgical castrated males and gilts [17]. However, more studies are required to investigate the relationship between sex and diet. The studies with a direct comparison of the sex type of pig and the legume used to feed pigs, for sensory quality traits on dry-cured loins, are scarce.

Regarding the coordinates of the different types of dry-cured loin and their correlation with sensory attributes (as shown in Figure 2), it can be observed that the CMS cured loin samples had a higher flavour persistence, acidity, and saltiness, and their fat appeared yellowish in colour. On the other hand, the CMP samples had a higher overall flavour intensity, with a sour taste and a darker (brown) muscle colour. The IMS and FS cured loins had a higher fat distribution and faecal odour, whereas the IMP and FP were juicier, sweeter, and had a stronger urine odour. Therefore, the trained panel’s sensory profiling of the dry-cured loin showed significant differences between the products from CMP and CMS, while the other treatments (IMP, IMS, FP, and FS) were not easily distinguishable. Our study found that pig immunocastration resulted in similar sensory properties in dry-cured loin treatments, reducing the differences between the two sexes. These findings could be related to Palma-Granados et al.’ [18] research on Iberian pigs’ backfat thickness at the last rib, where no differences were reported between IM and F, compared to CM, which had a higher fatness than the others. Differences in the fat content in cured pancetta from different sexes also showed differences in the sensory profile [19].

Table 3 shows the evaluation scores for various sensory attributes of dry-cured loins (*p*-values), along with the adjusted means by treatment. Among the evaluated attributes, the texture attribute of hardness was found to be the most discriminate, followed by fat colour, chewiness, and bitterness. The repeatability of the measured attributes indicates the ‘hardness descriptor’ varied slightly (r = 0.24) between panellists but met the acceptance criterion. This variation could be due to the small number of samples (three dry-cured loins) included in the experiment. On the other hand, the measurement of ‘fat distribution’ and ‘saltiness’ was found to be highly consistent across panellists (r = 0.88 and r = 0.76, respectively).

According to the literature, tenderness, juiciness, and flavour are the most important characteristics for meat quality [20]. These factors are influenced by the pig’s diet and sex. In dry-cured loins, the hardness, fat colour, bitterness, and chewiness (in 4 of the 16 attributes evaluated) were significantly affected (*p* < 0.01) by the pig’s diet and sex. However, there were no significant differences in texture, appearance, flavour intensity, and odour intensity (*p* > 0.05).

When pigs were fed a soya-based diet, the hardness values of dry-cured loin slices were affected by sex. The CMS dry-cured loin slices were less hard than for IMS and FS (CMS > IMS > FS). However, this effect was not observed when the pigs were fed a pea-based diet. Škrlep et al. [21] and Čandek-Potokar et al. [22] reported the same effect on sensorial hardness in dry-cured meat between surgical castrated male pigs and immunocastrated male pigs (immunocastrated female pigs were not studied). The differences in the amount of feed intake and lipogenic activity may explain the varying effects between sexes reported in another previous paper [23]. The study also found that IM pigs had a higher average daily feed intake but a lower feed conversion rate than CM pigs. This could have affected the results. However, the small sample size of the dry-cured loin may have masked the true potential for discrimination between the diets analysed. Additionally, the animal effect may have contributed to this.

Furthermore, the dry-cured loin slices from IMP were more tender than from CMP, with a less difficult chewiness. This could be due to the higher PUFA content in IMP, as shown in a previous study of Argemí-Armengol et al. [13]. The sex of the pig affects the lipogenic activity of their adipose tissues, which impacts the hardness and fatty acid profile [24]. The CMP cured loins had a higher bitterness than the IMP dry-cured loins. Bitter is a flavour descriptor related to the volatile organic compound benzaldehyde, which is classified within the chemical compound aldehydes, which originates from linoleic acid [25]. A previous article on fatty acids [13] reported linoleic acid content was higher in CMP than IMP, where the same raw loin material was used. Since surgically castrated male loins display a higher fat deposition compared with immunocastrated males, they could be more prone to lipolysis, yielding the aromas in this group. Żakowska-Biemans et al. [26] also reported that cured salami from IM pigs was more related to a sour flavour, whereas Čandek-Potokar et al. [22] reported no significant differences on bitterness between CM and IM in dry-cured ham.

The most outstanding attribute regarding appearance was the IMS loins which had the whitest fat colour, while CMS and FS loins had the yellowest. The yellow/brown colour of adipose tissues can result from oxidative deterioration, mainly the peroxidation of PUFA [20,27], and the accumulation of lipid oxidation products in cured pork products after long storage [28].

### 3.2. Consumer Panel

The second test involved evaluating consumer panel scores based on their gender, location, and consumption habits. Additionally, the sensitivity of consumers to skatole was tested using different pure substance concentrations. Non-significant single interactions were not reported in the text, as their *p*-value was greater than 0.05.

#### 3.2.1. Sensory Attributes of Dry-Cured Loins According to Socio-Demographics, Eating Patterns, and Skatole Sensitivity

Table 4 presents a summary of the outcomes of the questionnaire that was given to consumers regarding the sensory features of dry-cured loin samples, as well as their sociodemographic details, eating habits, and factors that influence their purchase of the loin. It is worth noting that the consumption pattern differed by region, which was not indicated in Table 4. Spanish consumers reported a higher consumption of dry-cured products than their Portuguese counterparts (77.3% vs. 65.0%, respectively, *p* < 0.001). According to FAO [29], the per capita pork consumption in Spain was 52 kg in 2020, while in Portugal it was 38 kg.

It was observed that individuals who did not show sensitivity to skatole had a higher age than those who claimed to perceive its unpleasant odour. The average age of individuals who did not perceive skatole’s unpleasant odour was 26.5 ± 0.6 years old compared to 23.7 ± 0.4 years old for those who could detect it at the threshold of 2 mg L^−1^ (*p* < 0.001). Similarly, individuals who could not detect skatole’s smell had an average age of 26.0 ± 0.5 years old, while those who could detect it at the 0.1–0.5 mg L^−1^ threshold had an average age of 24.2 ± 0.6 years old (*p* < 0.001). Font-i-Furniols et al. [30] suggested that age is correlated with sensitivity to odour scores of boar taint, especially androstenone odour. However, skatole was perceived by 99% of consumers and is therefore the main factor for the rating of flavour and odour in pork [31].

In terms of gender, a higher proportion of women showed sensitivity to skatole compared to men. Specifically, 66.7% of women had high-level sensitivity to skatole, while only 33.3% of men had it. Similarly, 67.9% of women had medium-level sensitivity to skatole, while only 22.1% of men had it. This difference was statistically significant (*p* < 0.001). However, in the current study, the proportion of people failing to claim undetectable skatole blindly was similar between genders (23.7% vs. 21.8%, *p* > 0.10). Škrlep et al. [21] found that almost all consumers were sensitive to skatole, with women generally being more sensitive than men. However, the study did not find any correlation between the acceptability of pork and the gender of consumers.

The study found that a higher percentage of consumers in Spain were sensitive to skatole compared to those in Portugal (71.9% vs. 63.8%, respectively; *p* < 0.01). On the other hand, a lower percentage of Spanish consumers showed medium skatole sensitivity compared to Portuguese consumers (41.7% vs. 57.5%, respectively; *p* < 0.001). Nevertheless, some studies suggests that the acceptance of pork and cluster segmentation is based more on the detection of boar taint than on the level of sensitivity to compounds [30,32].

The Spanish consumers rated the four sensory attributes (flavour, crumbliness, juiciness, and overall liking) lower than Portuguese consumers (*p* < 0.05). It is believed that these differences in the evaluation of consumer groups could be due to different liking expectations and the different loin standards of the region-of-origin assessors [33]. The results may also be influenced by the different method of organization of the test in the two regions (two sessions in Spain and one session in Portugal). However, these did not differ between genders (*p* > 0.05). Frequent consumers of dry-cured meat scored higher for the flavour, crumbliness, and overall liking of the evaluated dry-cured loins than less frequent consumers (*p* < 0.05). This finding is consistent with most cross-cultural consumer studies, where domestic or familiar products are more appreciated than international or unfamiliar products by consumers of a given culture [34]. Additionally, consumers who considered colour to be the main factor in their purchase of dry-cured loins rated higher on the evaluated sensory attributes than those who considered the marbling of dry-cured loins (*p* < 0.05). According to Morales et al. [35], colour is an aesthetic attribute that some consumers associate with a product of higher quality. The sensory attribute scoring did not differ between skatole sensitivity groups (*p* > 0.05). Although Font-i-Furnols et al. [30] showed that the acceptability of boar taint in pork increases when the frequency of cooking and eating fresh pork is higher, this finding varies depending on age groups.

#### 3.2.2. Consumer Sensory Assessment as Affected by Animal Diets and Sex

The results of consumer sensory trait scores for different treatments of dry-cured loins are presented in Table 5. The sensory evaluation conducted by consumers was influenced by the diet and sex of the animals (*p* < 0.05), particularly for flavour, juiciness, and overall liking. Crumbliness was not significantly affected (*p* > 0.05) by any of the different dry-cured loin treatments.

In general, the dry-cured loin samples from CMP and FP seemed to have higher liking scores, for a major part of sensory traits, than the other treatments (CMS, IMP, IMS, and FS), although globally they produced similar results in some sensory traits.

Consumers scored the dry-cured loin of CMP, IMP, and CMS as having more flavour (*p* < 0.05) than FS. According to a review by Lebret and Čandek-Potokar [36], pig sex strongly influenced the flavour attributes, but immunocastration in females and males reduced these effects. Moreover, the addition of 40% field pea in immunocastrated female pigs’ diet (at the end of the fattening period) positively influenced the sensory characteristics of dry-cured loin samples, resulting in higher flavour, juiciness, and overall liking (*p* < 0.05) than their counterparts fed a soya diet. The trained panel did not discriminate the flavour persistence and intensity among different treatments of dry-cured loin samples, but they perceived the CMP loins as being more bitter than IMP. The flavour and odour of cured pork were mainly determined by volatile compounds originating from lipid oxidative reactions, among others [37]. The literature describes that dry-cured Parma ham was more bitter when pigs were fed on a raw faba bean diet compared to a soy diet, while the raw pea diet had no effect on the bitter taste compared to the soy diet [38]. Likewise, dry-cured loins from pigs fed another local legume (Narbon vetch *Vicia narbonensis*), added at a concentration of 20% as a substitute for soybean, were perceived by consumers as having comparable sensory characteristics, but not more favourable [39].

According to the texture profiles, the dry-cured loin samples of surgical castrated male pigs (CM), immunocastrated male pigs (IM), and immunocastrated female pigs (F) had quite similar sensory properties related to crumbliness, which is consistent with other research on dry-fermented sausage and dry-cured bellies [26]. Additionally, F in raw cooked loin [40] also showed similar properties. Crumbliness and chewiness are closely related to the intramuscular fat content (IMF) [41], which confirms the results of homogeneity in the IMF from the different raw loin treatments reported in the previous paper [13].

In our study, the IM, CM (with both diets), and FP loin samples showed quite similar sensory juiciness traits, being different from FS. These findings in the consumers’ panel were not shown in the trained panel. Further studies should be carried out to confirm these results. A study has shown that the sensory properties of meat products influence consumer taste [20] and determine their success or failure on the food market [42].

Finally, consumers’ overall assessment of dry-cured loin clearly scored the most valued as the CMP and the worst valued as the FS (*p* < 0.05), but they did not discriminate among the other treatments. Therefore, there were no significant differences among the other treatments. This suggests that the sensory quality of cured loin from pigs fed with pea is not inferior to that from pigs fed with a soya diet. Therefore, it is recommended to use locally produced feed ingredients to reduce the dependence on imported soy [43].

## 4. Conclusions

In this preliminary study, it was observed that trained panellists were able to distinguish between dry-cured loins obtained from pigs fed a field pea-based diet and those fed a soya-based diet when the male pigs had been surgically castrated, but not when the pigs were immunocastrated (male and female pigs). The pea-based diet seems to improve the chewiness and reduce the bitterness of dry-cured loins from immunocastrated male pigs. Additionally, loins from surgical castrated male pigs fed a pea-based diet resulted in tougher and more bitter meat than those from immunocastrated male pigs. Interestingly, immunocastration did not compromise the skatole detection by trained panellists.

Although the Spanish and Portuguese have different consumption patterns for dry-cured loin, this study seems to have found that most sensory attributes performed better in dry-cured loins from surgical castrated male pigs and immunocastrated female pigs that were fed peas. It is important to conduct further research on the sensory properties of cured meat products based on the relationship between the sex of the animal and its diet. Therefore, home-grown field pea as a protein source in feed and immunocastration may be good alternatives to providing cured meat products that align with sustainability labelling commitments.

## Figures and Tables

**Table 1:** Residual variance, scaling factors, and percentage variation explained by the first two principal components for each assessor for dry-cured loin sensory analysis.

Panellists	Residual	Scaling Factors	F1	F2
P1	3.8327	0.6141	14.8958	17.2396
P2	2.3431	0.8845	14.0750	50.7443
P3	1.9992	1.8457	51.1693	14.4044
P4	3.5848	0.9223	27.7374	13.1265
P5	1.4392	0.9956	40.7184	17.9005
P6	1.9579	1.1541	26.0096	22.1436
P7	2.4336	1.1288	34.2492	18.1800
P8	1.0139	1.5098	27.7532	31.8875

F1, F2: two first principal components.

**Table 2:** Results from principal component analysis of the taste panel evaluations.

	F1	F2	F3	F4	F5
Eigenvalue	0.597	0.491	0.399	0.270	0.239
Variability (%)	29.932	24.614	19.994	13.505	11.955
Cumulative %	29.932	54.546	74.540	88.045	100.000

F1, F2, F3, F4, F5: five first principal components.

**Table 3:** Mean scores of sensory attributes of dry-cured loins, evaluated by trained panel (ranked from 1 to 9, from least to most liking).

Attributes	IMS	IMP	CMS	CMP	FS	FP	SEM	*p*-Values	Repeatability (r)
Hardness (tender to tough)	4.38 ab	3.65 bc	3.38 c	4.40 a	4.15 ab	3.94 abc	0.251	***	0.24
Fat colour (white to yellow)	2.45 b	2.63 ab	3.00 a	2.90 ab	3.13 a	2.75 ab	0.188	***	0.31
Chewiness (easy to difficult)	4.07 ab	3.44 b	3.50 ab	4.25 a	4.00 ab	3.90 ab	0.284	**	0.24
Bitter (low to high)	1.88 ab	1.50 b	1.69 ab	2.19 a	1.73 ab	1.71 ab	0.179	**	0.38
Saltiness (low to high)	5.29	5.63	5.50	5.69	5.21	5.35	0.259	ns	0.76
Juiciness (dry to juicy)	4.67	5.31	5.25	4.65	4.75	5.06	0.286	ns	0.54
Fat distribution (homogeneous to focalized)	6.65	6.69	6.15	6.67	6.56	6.56	0.339	ns	0.88
Flavour persistence (low to high)	5.49	5.52	5.87	5.77	5.65	5.79	0.295	ns	0.73
Acidity (low to high)	1.78	1.88	2.06	2.00	1.90	1.71	0.160	ns	0.14
Muscle/fat ratio (fat to muscle dominance)	7.32	7.31	7.27	7.15	7.27	7.02	0.170	ns	0.33
Do you detect androstenone, urine odour? (low to high)	1.48	1.38	1.42	1.19	1.23	1.48	0.158	ns	0.18
Muscle colour (lean) (picture grading)	4.56	5.48	4.83	5.40	5.02	4.58	0.430	ns	0.33
Sweetness (low to high)	2.37	2.29	2.54	2.35	2.40	2.54	0.215	ns	0.59
Do you detect skatole, faeces odour, stable odour? (low to high)	1.42	1.21	1.17	1.15	1.19	1.19	0.145	ns	0.16
Flavour intensity (overall assessment) (low to high)	5.98	5.83	6.00	6.06	5.85	5.90	0.253	ns	0.70
Odour intensity (low to high)	5.98	6.04	5.88	5.96	5.75	5.92	0.245	ns	0.61

SEM = standard error of the mean. Different letters within each row denote statistical differences between treatments. *** (*p* < 0.001), ** (*p* < 0.01), ns (*p* > 0.05). IMS: immunocastrated male pigs with a soya diet; IMP: immunocastrated male pigs with a pea diet; CMS: surgical castrated male pigs with a soya diet; CMP: surgical castrated male pigs with a pea diet; FS: immunocastrated female pigs with a soya diet; FP: immunocastrated female pigs with a pea diet. (r) Repeatability was estimated from the trained panel (Vt) and residual (Vr) components of the variance of aleatory effects, using the following formula: r = (Vt/(Vt + Vr)).

**Table 4:** Mean scores of sensory attributes of dry-cured loins according to consumer location, gender, eating pattern, determinant purchasing cues, and sensitivity to skatole (ranked from 1 to 5, from least to most liking).

		Flavour	Crumbliness	Juiciness	Overall Liking
Location (L)	Spain	3.39	3.36	3.41	3.38
	Portugal	3.73	3.86	3.82	3.73
Gender (G)	Woman	3.51	3.58	3.56	3.5
	Man	3.61	3.64	3.68	3.62
Eating pattern of dry-cured sausages (P)	≤once/month	3.42	3.50	3.55	3.44
	≥once/week	3.70	3.72	3.69	3.67
Main purchasing driver (D)	Colour	3.63	3.71	3.70	3.65
	Marbling	3.49	3.52	3.54	3.47
Skatole sensitivity <2 mg/L (Sen 2)	Yes	3.48	3.53	3.57	3.47
	No	3.64	3.69	3.67	3.64
Skatole sensitivity <0.1–0.5 mg/L (Sen 1)	Yes	3.56	3.57	3.65	3.59
	No	3.56	3.65	3.59	3.52
Failed to assign blank (Sen 0)	Yes	3.55	3.7	3.65	3.55
	No	3.57	3.52	3.59	3.56
SEM		±0.071	±0.072	±0.069	±0.069
*p*-value	L	***	***	***	***
	G	ns	ns	ns	ns
	P	**	*	ns	**
	D	ns	*	*	*
	Sen 2	ns	ns	ns	ns
	Sen 1	ns	ns	ns	ns
	Sen 0	ns	ns	ns	ns

SEM = standard error of the mean. *** (*p* < 0.001), ** (*p* < 0.01), * (*p* < 0.05), ns (*p* > 0.05).

**Table 5:** Mean scores of sensory traits of dry-cured loins evaluated by the consumer panel (ranked from 1 to 5, from least to most liking).

Descriptors	IMS	IMP	CMS	CMP	FS	FP	SEM	*p*-Values
Flavour	3.39 bc	3.45 bc	3.71 ab	3.90 a	3.19 c	3.74 ab	0.10	*
Crumbliness	3.50	3.70	3.50	3.93	3.21	3.77	0.10	ns
Juiciness	3.56 ab	3.63 a	3.70 a	3.90 a	3.19 b	3.73 a	0.10	*
Overall liking	3.4 5 bc	3.51 bc	3.62 b	3.97 a	3.13 c	3.67 ab	0.10	*

SEM = standard error of the mean. Different letters within each row denote statistical differences between treatments. * (*p* < 0.05), ns (*p* > 0.05). IMS: immunocastrated male pigs with a soya diet; IMP: immunocastrated male pigs with a pea diet; CMS: surgical castrated male pigs with a soya diet; CMP: surgical castrated male pigs with a pea diet; FS: immunocastrated female pigs with a soya diet; FP: immunocastrated female pigs with a pea diet.

## Data Availability

The data that support the findings of this study are available from the corresponding author, I.A.-A., upon reasonable request.
